# Enzyme Commission Number Prediction and Benchmarking with Hierarchical Dual-core Multitask Learning Framework

**DOI:** 10.34133/research.0153

**Published:** 2023-05-31

**Authors:** Zhenkun Shi, Rui Deng, Qianqian Yuan, Zhitao Mao, Ruoyu Wang, Haoran Li, Xiaoping Liao, Hongwu Ma

**Affiliations:** ^1^ Biodesign Center, Key Laboratory of Engineering Biology for Low-carbon Manufacturing, Tianjin Institute of Industrial Biotechnology, Chinese Academy of Sciences, 300308, Tianjin, China.; ^2^ National Center of Technology Innovation for Synthetic Biology, 300308, Tianjin, China.; ^3^ College of Biotechnology, Tianjin University of Science & Technology, Tianjin, China.; ^4^ Haihe Laboratory of Synthetic Biology, 300308, Tianjin, China.

## Abstract

Enzyme commission (EC) numbers, which associate a protein sequence with the biochemical reactions it catalyzes, are essential for the accurate understanding of enzyme functions and cellular metabolism. Many ab initio computational approaches were proposed to predict EC numbers for given input protein sequences. However, the prediction performance (accuracy, recall, and precision), usability, and efficiency of existing methods decreased seriously when dealing with recently discovered proteins, thus still having much room to be improved. Here, we report HDMLF, a hierarchical dual-core multitask learning framework for accurately predicting EC numbers based on novel deep learning techniques. HDMLF is composed of an embedding core and a learning core; the embedding core adopts the latest protein language model for protein sequence embedding, and the learning core conducts the EC number prediction. Specifically, HDMLF is designed on the basis of a gated recurrent unit framework to perform EC number prediction in the multi-objective hierarchy, multitasking manner. Additionally, we introduced an attention layer to optimize the EC prediction and employed a greedy strategy to integrate and fine-tune the final model. Comparative analyses against 4 representative methods demonstrate that HDMLF stably delivers the highest performance, which improves accuracy and F1 score by 60% and 40% over the state of the art, respectively. An additional case study of tyrB predicted to compensate for the loss of aspartate aminotransferase aspC, as reported in a previous experimental study, shows that our model can also be used to uncover the enzyme promiscuity. Finally, we established a web platform, namely, ECRECer (https://ecrecer.biodesign.ac.cn), using an entirely could-based serverless architecture and provided an offline bundle to improve usability.

## Introduction

With the widespread adoption of high-throughput methods and high-quality infrastructure, the speed of new protein discovery has increased dramatically. However, this was not followed by a concomitant increase in the speed of protein annotation. For example, 801,118 sequences were added to TrEMBL in the UniProt database [1] in the single month of December 2022, while only 388 sequences were reviewed and added to Swiss-Prot in the same period (Fig. S2). Such a slow speed of protein annotation considerably restricts related research and industrial applications.

Among the multiple and complex protein annotation tasks, one of the crucial steps is enzyme function annotation [2,3]. Annotations of enzyme function provide critical starting points for generating and testing biological hypotheses [3]. Current functional annotations of enzymes describe the biochemistry or process by assigning an enzyme commission (EC) number. An EC number is a 4-part code associated with a recommended name for the corresponding enzyme-catalyzed reaction that describes the enzyme class, the chemical bond acted on, the reaction, and the substrates [4]. Thus, the primary task of enzyme annotation is to assign an EC number to a given protein sequence. However, as the uncertainty of the assignments for uncharacterized protein sequences is high and biochemical data are relatively sparse, both the speed and the quality of enzyme annotation are considerably restricted.

To achieve improved, rapid, and intelligent functional annotation, computational methods were introduced to assign or predict EC numbers. The simplest and most commonly used method is based on sequence homology [5]; researchers have developed a variety of EC databases and profile-based methods for the functional annotation of enzymes [6–8]. However, these methods cannot perform annotations for novel proteins without similar sequences, which is generally the case for newly discovered enzymes. To overcome this restriction, researchers introduced machine learning (ML) methods, such as hidden Markov model [9], K-nearest neighbor (KNN) [10], and SVM [11] for annotating enzymes. Although these methods can predict EC numbers for proteins without similar references, the prediction speed and precision are not ideal. Because deep learning has delivered powerful results in many areas [12–15], researchers use deep learning methods to predict EC numbers and continually improve the precision of functional annotation [2]. However, deep learning methods are prone to overfitting because of an unbalanced distribution of training datasets [16]. Specific to the EC number prediction task, this would lead to predictions with high precision, medium recall, and low accuracy.

Overall, there has been a steady improvement in computational methods for enzyme annotation [2,7,17,18], but several obstacles still exist that have slowed the progress of computational enzyme function annotation. The first is a lack of publicly available benchmark datasets to evaluate the existing and newly proposed models, making it troublesome for the end user to choose the best method in their production scenario. Another hindrance is the lack of an explicitly designed method with stable prediction performance to deal with newly discovered proteins. Especially, the lacking of an efficient and universal protein sequence embedding method made researchers have to spend large amounts of time on handcrafted feature engineering to encode the sequence, such as functional domain encoding [19] and position-specific scoring matrix (PSSM) encoding [20], as encoding quality dramatically affects the performance of downstream applications [21]. The third one is the usability of existing tools, as some tools are only available offline and are not easy to install, while some online tools are no longer available.

To overcome these obstacles, we proposed a hierarchical dual-core multitask learning framework (HDMLF) in this work. The main contributions are as follows:

• We constructed 3 standard datasets for benchmarking and evaluation. The datasets contain more than 470,000 distinct labeled protein sequences from Swiss-Prot.
• We proposed a novel framework on the basis of the latest protein language embedding methods and gated recurrent unit (GRU) with the attention mechanism. In HDMLF, we formulate the EC number prediction as a multitask multilabel classification problem. The first task predicts whether a given protein sequence is an enzyme. The second task predicts how many functions the enzyme can perform, i.e., multifunctional enzyme prediction. The last task predicts the exact EC number for each enzyme function. To achieve cutting-edge performance for EC number prediction, we first introduced and evaluated the state-of-the-art deep learning language embedding methods for universal protein sequence embedding [22,23]. Then, a novel prediction method based on GRU with an attention mechanism was proposed to solve the 3 tasks in a multitasking manner. Finally, a feedback mechanism is adopted to choose the most suitable embedding, and a greedy strategy is introduced to integrate these tasks to maximize the EC prediction performance.
• A webserver was published for easy usability. We published a web platform based on a serverless architecture so that anyone can annotate EC numbers smoothly in high throughput, whether they have coding experience or not. Our webserver is publicly accessed via http://ecrecer.biodesign.ac.cn.

## Results

### A dataset for benchmarking

Because of the lack of a public benchmark for EC number prediction, we constructed a standard dataset from Swiss-Prot for model development and evaluation. To simulate real application scenarios as closely as possible, we organized data chronologically. Specifically, we used a snapshot from February 2018 as the training dataset, consisting of 469,134 records (4,854 distinct EC numbers). We construct 2 testing sets to simulate the real protein discovery and annotation processes and validate the EC prediction performance of effectiveness and stability with time variance. Testing set 1 (testset_20) is from the June 2020 snapshot, consisting of 7,101 records (937 distinct EC numbers). Testing set 2 (testset_22) is from February 2022, consisting of 10,614 records (1,355 distinct EC numbers). All testing sets filtered the sequences that appeared in the training set. The details are listed in Tables S2 to S5.

### Suitable embedding methods do help in improving the prediction performance

ML models trained on protein sequences and their measured functions can infer unseen sequences’ biological properties without understanding the underlying physical or biological mechanisms. However, ML models require vectors as input other than amino acid sequences, and converting from a protein sequence to a vector representation extremely affects the model’s ability to learn [21].

To evaluate and choose the best embedding methods for our downstream prediction tasks, we evaluated 3 different protein embedding methods, one-hot embedding, UniRep embedding, and evolutionary scale modeling embedding method (ESM) embedding with different layers (from 1 to 33) in 2 different testing datasets. The evaluation process can be categorized into 2: (a) We use an enzyme or non-enzyme prediction task and 6 ML baselines to evaluate the embedding performance in binary classifications, and (b) we use the EC prediction task and our proposed method to evaluate the embedding performance in multiclass classifications. The 6 ML baselines are KNN, logistic regression (LR), XGBoost, decision tree (DT), random forest (RF), and gradient boosting decision tree (GBDT).

• Protein embedding methods can learn semantic information from sequences directly, remarkably improving downstream tasks’ performance.

**Fig. 1.**
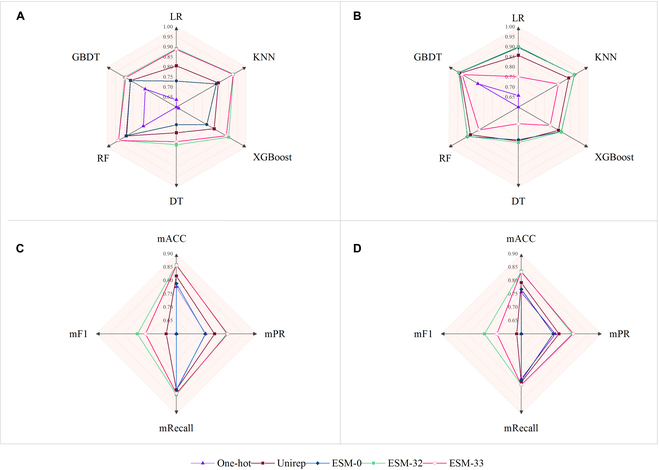
Performance comparison of different embedding methods. (A and B) Embedding performance (F1 score) comparison using ML baselines on task 1. (A) Results on testset_20 and (B) on testset_22. (C and D) Embedding performance comparison using our proposed framework HDMLF on task 3; mACC, mPR, mRecall, and mF1 are different evaluation metrics defined in the “Evaluation metrics” section. (C) Results on testset_20 and (D) on testset_22.

As shown in Fig. 1, compared with the traditional one-hot protein representation, methods UniRep and ESM improve the downstream task by more than 20% in all testing tasks, in terms of F1 score (details can be found in Tables S6 and S7). For embedding, ESM-32 exhibited the best overall performance among all 6 baselines regarding all evaluation metrics for embedding. As shown in Fig. 1C and D, in the EC prediction task, ESM-32 achieved 21.67% and 6.03% improvements over one-hot and UniRep in terms of accuracy, as well as 27.20% and 7.32% in terms of mF1, respectively. This experiment suggests that better embedding can lead to better learning performance, and deep latent representation can comprehensively represent the protein sequence. Apart from protein sequences, protein structure can also be helpful in conducting protein embedding, and existing studies have demonstrated this [24,25]. In our future work, we plan to incorporate these techniques to further enhance the prediction performance.

• For embedding layers, not the deeper the better.

To validate the effectiveness within different depths of embedding layers, we evaluated embedding layers 1 to 33 from ESM under 4 classification baseline methods. Interestingly, we found that, when layers increase from 1 to 32, the performance increases, while when layers reach 32, the performance began to decrease (Fig. 1); this is mainly due to the overfitting issue and suggests that, for selection of embedding layers, not the deeper, the better.

### HDMLF versus existing EC prediction methods

To achieve the best performance, HDMLF is proposed with 3 objectives: (a) classify the enzyme and non-enzyme with high accuracy, (b) provide the ability to predict multifunctional enzymes, and (c) achieve state-of-the-art EC prediction performance, and we treat these objectives as 3 learning tasks. To meet these objectives, our proposed framework is designed with mutitask learning techniques and is organized in a hierarchical order.

To evaluate the performance of HDMLF and validate whether these objectives do help our final goal, we made a comprehensive comparison experiment with existing EC prediction methods among all these tasks.

#### EC number prediction performance comparison

**Fig. 2.**
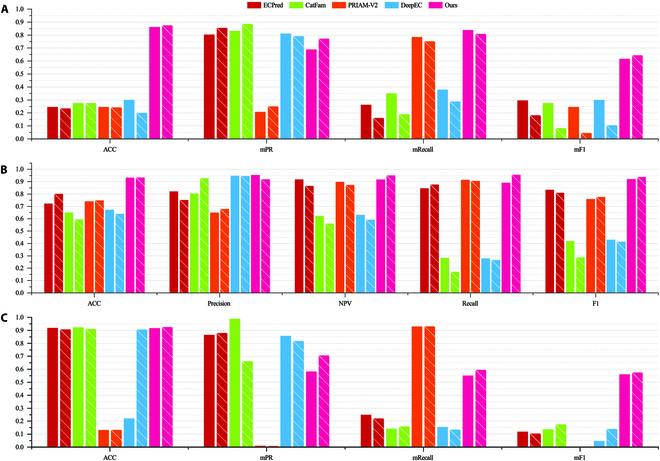
Performance comparison of different methods for different prediction tasks. Bars with 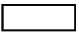 represent prediction results on testset_20, bars with 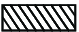 represent prediction results on testset_22 (A) Performance comparison on EC number prediction. (B) Performance comparison on enzyme and non-enzyme prediction. (C) Performance comparison on task 2 multifunctional enzyme prediction.

EC prediction is the main task and final goal in HDMLF. As shown in Fig. 2C, our proposed methods achieved the best overall performance in testset_20. PRIAM [7] is mainly designed to include more sequences, so the mRecall is high (78.48%, 75.26%), while the mPR (20.80%, 25.03%) is very low. DeepEC, ECPred, and CatFam pursue high precision; these methods are very likely to miss many new functions, which, in turn, wound underperform in terms of accuracy and F1 score. The F1 score should be a better evaluation metric for the EC assignment of real-world proteins. In terms of mF1 score, our model reached over 150% performance than the second best method, which achieved 86.91% accuracy with 69% mPR and 63.88% mRecall. In other words, if 100 protein sequences were uploaded for annotation, then we can obtain approximately 87 correct annotations. Interestingly, existing methods show much poorer performance on testset_22 while our method can maintain the prediction performance. We conducted further validation of the HDMLF’s performance in predicting EC numbers at first to third levels compared to other baselines. The results are shown in Tables S11 to S13. Our findings demonstrate that the HDMLF outperforms the second-best method by more than 20%, 28%, and 30% in terms of mF1 at the first, second, and third levels of EC, respectively. However, when we simplify the evaluation criteria and only consider the first-digit EC, the mF1 score remains below 70%. Note that these methods were specifically designed to predict the fully 4-digit EC number, which is not optimal for first-digit EC prediction.

Overall, in testset_22, for these relatively new proteins, our method much improved the mF1 score over existing methods; this is mainly due to the EC numbers and enzyme samples being more inclusive in testset_22. All the above results show that our method shows a clear advantage in terms of EC number assignment.

#### Enzyme or non-enzyme prediction performance comparison

In enzyme or non-enzyme prediction task, as shown in Fig. 2B, our method can achieve scores of 92.01% and 93.61% in terms of F1 score in the above-constructed testing sets, respectively. Compared with other state-of-the-art tools, the overall accuracy was greatly improved. Many previous methods were designed to obtain high precision while neglecting accuracy, negative predictive value, and recall. For example, DeepEC can reach 94.68% precision while recall is only 20.83% in testset_20, which means that many enzymes would be missed by DeepEC prediction.

#### Multifunctional enzyme prediction

The multifunctional enzyme prediction task is designed to predict the number of ECs assigned to a given protein. As shown in Fig. 2C, we can see that the performance of our proposed method is stable and superior to existing baselines. In testset_20, our method achieved 91.71% accuracy with 58.37% mPR and 55.20% mRecall recall. In testset_22, our method achieved 92.45% accuracy with 70.68% mPR and 59.56% mRecall recall. The low mRecall and mPR are mainly due to the data sparseness of 3 to 8 functional enzymes , which results in the classifier being more preferred to predict an enzyme as a single-function enzyme. Although our proposed method achieved the best performance among existing methods, this is still inadequate in a productive scenario, especially for enzymes assigned with more than 3 EC numbers, so it should be further improved in future work.

### Assessing the stability of EC prediction performance over time

**Fig. 3.**
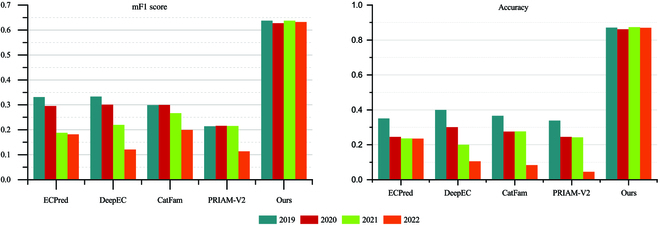
Task 1 comparison of EC prediction performance over time.

To assess the efficacy and predictability of our proposed framework over time, we simulated an EC prediction experiment using Swiss-Prot data in a realistic setting. We first collected the snapshot of Swiss-Prot from 2019 to 2022. Then, we excluded the data that appeared in the training set and used the trained model to predict the new data added from 2019 to 2022. Comparative results with existing EC prediction methods are shown in Fig. 3. From Fig. 3, we can observe that all methods’ prediction performance will decrease over time. In terms of baselines, DeepEC and CatFam are relatively more stable than the others. For DeepEC, the performance in terms of F1 score dropped by 3.20%, 11.34%, and 21.20% from 2020 to 2022, compared to 2019, and decreased by 9.86%, 19.94%, and 29.48% in terms of accuracy. The reason for this is that DeepEC uses one-hot embedding and 3 different models to predict the different levels of EC, and it integrates the output using the sequences alignment method. While the first level of EC prediction is much more precise, one-hot embedding cannot preserve sufficient information to represent protein sequences. Moreover, it is weak in the fourth level of EC prediction, and the sequence alignment method cannot handle new sequences with low similarities. On the other hand, CatFam’s performance dropped by −0.4%, 3.28%, and 9.95% from 2020 to 2022, compared to 2019, in terms of F1 score and decreased by 9.02%, 8.99%, and 28.25% in terms of accuracy. This is because CatFam is based on homologous similarity and PSSM. It is more stable for protein sequences with existing homologous sequences than DeepEC. However, the performance will decrease significantly for newly added proteins without homologous sequences. Remarkably, the prediction performance of our proposed method is more stable, decreasing by less than 1% from 2019 to 2022, both in terms of F1 score and accuracy. This mainly benefits from the language model, which is powerful in sequence embedding and can reserve sufficient information for downstream applications, and we treat 4-level EC as a tuple that reduces the integration error from different predictive models. Thus, our proposed method is considerably more statable than the existing ones.

### Case study: HDMLF can annotate protein with incomplete EC number

**Fig. 4.**
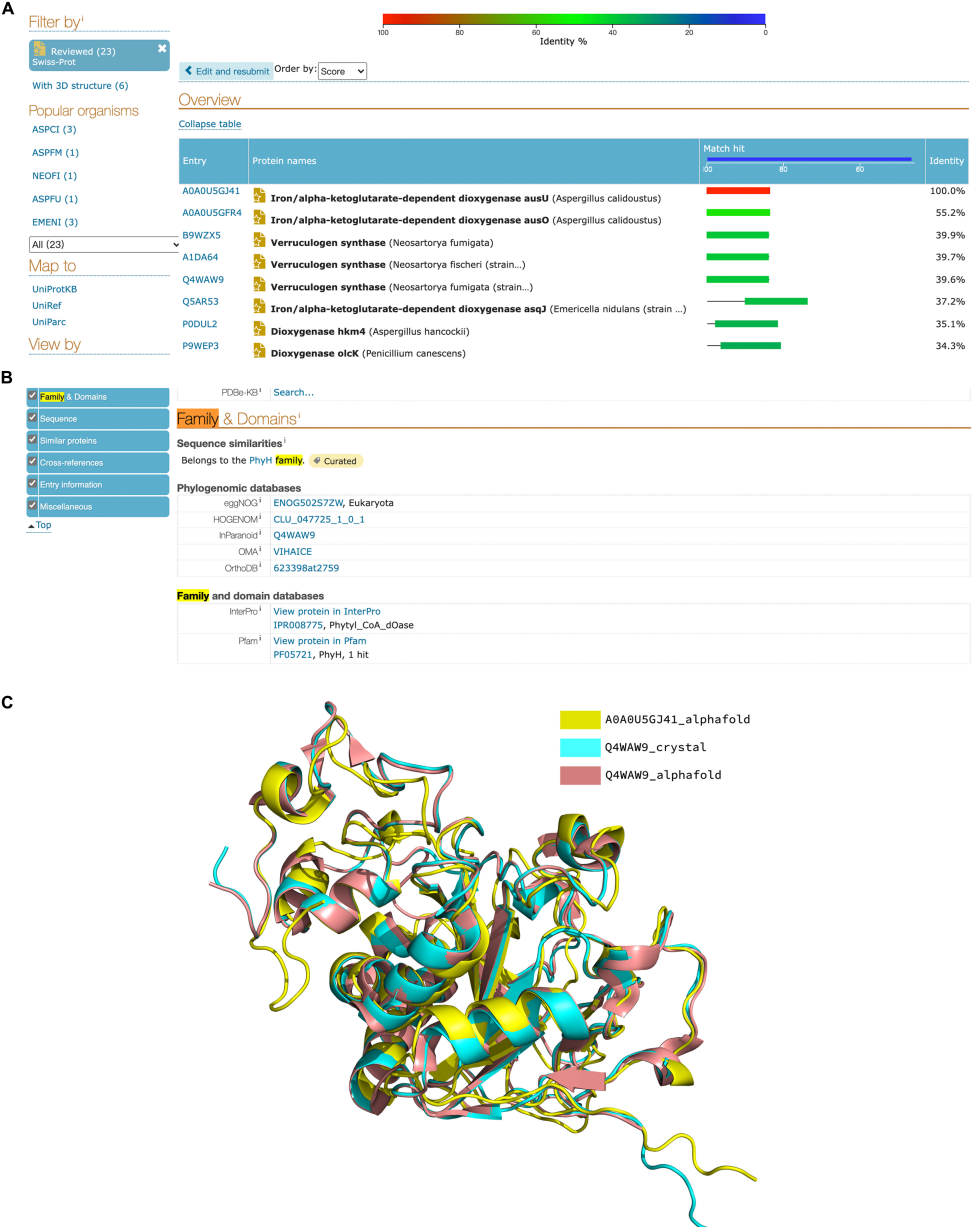
(A to C) Comparison of sequence similarity and structural similarity.

In the databases, many enzymes with EC numbers exist in an uncompleted 3-level, 2-level, or even 1-level state. These proteins with incomplete EC numbers might not directly be utilized for retrieving enzymatic reactions. For instance, an enzyme iron/alpha-ketoglutarate-dependent dioxygenase AusU (UniProt ID: A0A0U5GJ41) has a 2-level EC number in the database (1.14.-.-), while our method HDMLF can assign this protein with the fourth-level EC number 1.14.11.38. After blasting it against the UniProt database, we find that the top 5 reviewed proteins with the highest identities include 3 verruculogen synthases (Fig. 4A). Because only Q4WAW9 has a crystal structure, we take protein Q4WAW9 [26] as an example and find that both genes belong to exactly the same protein families with the same domains (Fig. 4B). To further validate the results, we compare the structure of A0A0U5GJ41 (alphfold2 predicted) and Q4WAW9 (alphfold2 predicted and crystal structure). The results show that these 2 proteins have a highly similar structure (Figs. S4 to S7) with a small root mean square deviation (1.104). Because Q4WAW9 has a 4-level EC number 1.14.11.38, the protein A0A0U5GJ41 could be potentially annotated as EC 1.14.11.38 as well, which supports our 4-level prediction.

### Case study: HDMLF can uncover enzyme promiscuity

Enzyme promiscuity toward substrates has been discussed in evolutionary terms as providing the flexibility to adapt to novel environments. Moreover, it has been demonstrated that many enzymes exhibit flexibility, or promiscuity, in regard to what substrates their catalytic pockets recognize. A previous study using model-driven approaches found that even in *Escherichia coli*, a well-studied model species, many underground reactions still occur [27]. For example, gene essentiality analysis and gene knockout experiment revealed that gene tyrB, which is annotated as tyrosine aminotransferase (EC number 2.6.1.57; 2.6.1.107 in UniProt), also has aspartate aminotransferase (encoded by aspC; EC number 2.6.1.1 in UniProt) activity and thus can compensate for aspC gene deletion [28]. The knockout of the gene encoding the potential isozyme revealed that tyrosine aminotransferase, which is encoded by tyrB (EC number 2.6.1.57; 2.6.1.107 in UniProt), can compensate for the loss of aspartate aminotransferase, which is encoded by aspC (EC number 2.6.1.1 in UniProt).

**Fig. 5.**
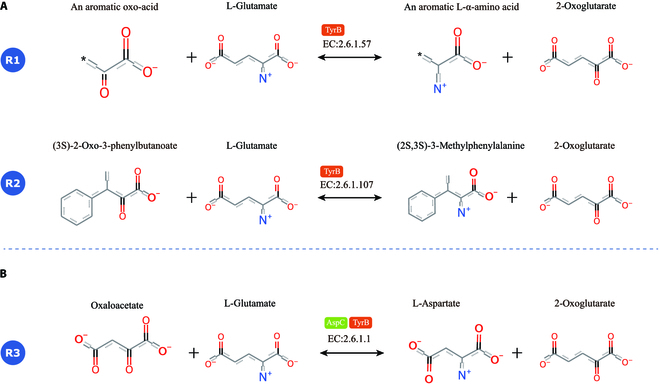
(A) TryB is assigned with EC numbers 2.6.1.57 and 2.6.1.107 in UniProt. (B) Our model can assign TyrB with additional EC number 2.6.1.1, and this is consistent with a previous experimental study in *Escherichia coli*, which finds that tyrB can compensate for the loss of aspC (EC number 2.6.1.1 in UniProt), while DeepEC can only assign tyrB with EC number 2.6.1.57.

Although the specific functional annotation and substrate are different, our method can uncover the underground reaction 2.6.1.1 for tyrB (Fig. 5C) . In addition, our method can also assign EC numbers 2.6.1.57 and 2.6.1.107 to tryB. However, with DeepEC, only 2.6.1.57 can be assigned. This can be further validated by the EcoCyc [29], a well-annotated database for *E. coli* MG1655, as tryB was assigned with EC number 2.6.1.1 in EcoCyc. In addition, there are 852 proteins with more than 90% similarity with tyrB in the UniProt database based on a homology search. Only one protein(UniProt ID: P74861) was reviewed and the assigned EC number is 2.6.1.57. All the other proteins were assigned with EC number 2.6.1.-. Because these proteins all have very high similarity with tryB, they should all have the EC number 2.6.1.1. As expected, our model can assign these proteins with EC number 2.6.1.1. All these cases show that our model can be used to uncover enzyme promiscuity.

### Case study: HDMLF can uncover unknown enzyme for a not well-annotated genome

To demonstrate the inclusiveness and predictive ability of our proposed method, we conducted EC number prediction on unreviewed proteins. *Corynebacterium glutamicum*, the famous industrial workhorse for amino acid production with a current output of over 6 million tons per year [30], is increasingly being adopted as a promising chassis for the biosynthesis of other compounds. However, unlike *E. coli* (1,652 protein sequences with EC numbers out of 4,322 proteins, 38.2%), the protein sequences of *C. glutamicum* were not well annotated. Of 3,305 protein sequences, only 537 were reviewed and included in the Swiss-Prot database (357 proteins have assigned EC numbers). We used the other 2,768 protein sequences to compare our tool with DeepEC. Our approach was able to assign 1,056 proteins with EC numbers, while DeepEC only assigned 157 EC numbers (123 same EC numbers between DeepEC and HDMLF). We found many interesting cases, for example, pyrimidine reductase, an enzyme involved in riboflavin biosynthesis (UniProt ID: Q8NPB8); DeepEC and TrEMBL both predict it to be non-enzyme, which is not correct, while our model predicts it with EC number 1.1.1.302 with high confidence. Another case is deoxynucleoside monophosphate kinase (UniProt ID:Q8NPP0); DeepEC and TrEMBL cannot assign an EC number, while our model predicts it with EC number 2.7.4.13. As shown in the Rhea database (RHEA:11216), the corresponding reaction and enzyme class are all consistent with protein annotation. These results again show that our method can better annotate new proteins than existing annotation tools.

### ECRECer: A web platform for EC prediction based on HDMLF

To enhance the usability of our proposed framework (Fig. S8) so that the end user can use them smoothly even with no coding experience, we built a web application using a cloud-based architecture, offering high reliability, robustness, and scalability. The static files such as the HTML pages and JavaScript codes are hosted on AWS S3 and distributed through AWS CloudFront for speed of content delivery to users worldwide. End users can simply upload sequences to our platform and then click the submit button to trigger the prediction workflow. AWS API Gateway routes HTTP requests from the front end to the backend. We used AWS Step Functions to coordinate the components of our applications, process messages passed from AWS API Gateway, and invoke the workflows asynchronously. We used AWS S3 to exchange data between jobs and store the result files. In general, the whole workflow can be completed in a few seconds. We use Amazon DynamoDB to store job information, and users can track their previous submission records and corresponding status information. Once the analysis is finished, the user can view or download the corresponding results.

## Conclusion

In this work, we proposed a novel HDMLF to complete 3 benchmarking tasks: (a) enzyme or non-enzyme annotation, (b) quantity of EC numbers prediction, and (c) EC number prediction. The method developed in this work has 2 cores, an embedding core and a learning core. The embedding core is responsible for selecting the best available embedding method among one-hot, UniRep, and ESM to calculate sequence embeddings. The learning core is responsible for completing the specific benchmarking tasks using the best-calculated protein sequence embedding as input in a hierarchical multitask way.

We were guided by 2 principles in the design of HDMLF. The first principle is providing state-of-the-art EC number prediction performance. The second principle is high usability (both can be accessed via the world wide web and offer a standalone suit for high-throughput prediction). To implement the first principle, we proposed HDMLF, which integrates the protein language model with a hierarchical BGRU with an attention mechanism. To implement the second principle, we provided a web server (ECRECer) and a standalone package. We opened all the source codes, including data preprocessing, dataset buildup, model training, and model testing/evaluation.

Comprehensive comparisons with existing state-of-the-art methods demonstrated that our method is highly competitive and has the best performance with high usability. In addition, our method can be used to uncover enzyme promiscuity. Although our method exhibited the best performance, it still needs improvement. For example, the performance of multifunctional enzyme annotation is relatively low, while the accuracy and recall of EC number annotation are less than 90%.

**Table:** 

Key points: • An HDMLF framework is proposed to predict EC numbers by using protein sequence data. • A protein language model and an extreme multilabel classifier are adopted to reduce the heavy head-crafted feature engineering and elevate the prediction performance. • The proposed framework remarkably outperforms the existing state-of-the-art method in terms of accuracy and mF1 score by 70% and 20%, respectively. • An online service and an offline bundle are provided for end users to annotate EC numbers in high throughput easily and efficiently.

## Materials and Methods

### Problem formulation

To annotate the enzyme function of a new protein sequence, the initial and basic task is to define whether the given protein is an enzyme. Because there are numerous multifunctional enzymes, the next task is to determine the quantity of EC numbers. After completing the above 2 tasks, it is necessary to assign an EC number to each function. On the basis of these considerations, we proposed 3 basic tasks for the functional annotation of enzymes, as shown below.

#### Enzyme or non-enzyme annotation

The enzyme or non-enzyme annotation task is formulated as a binary classification problem:

$$f:X\to \left\{0,1\right\}$$ (1)

where *X* = {*x*_1_, *x*_2_, ⋯, *x_n_*}, *n* ≥ 1 represents a group of protein sequences, and {0, 1} is the label indicating whether a given protein is an enzyme.

#### Multifunctional enzyme annotation

Multifunctional enzyme annotation is formulated as a multiclassification problem:

$$f:X\to \left\{1,2,\cdots ,k\right\},$$ (2)

where *k* represents the maximum number of EC numbers for a given protein.

#### EC number assignment

The EC number assignment task is also formulated as a multiclassification problem as defined in Eq. 3.

$$f:X\to \left\{1.1.1.1,1.1.1.2,\cdots \right\},$$ (3)

### Dataset description

To address the first challenge, we constructed 3 standard datasets (Supplementary Materials). Similar to previous work [21,25], these datasets are extracted from the Swiss-Prot database. To simulate real application scenarios as closely as possible, we did not shuffle data randomly. Instead, after data preprocessing (Supplementary Materials), we organized data in chronological order. Specifically, we used a snapshot from February 2018 as the training dataset. To simulate the real protein discovery and annotation processes and validate the EC prediction performance of effectiveness and stability with time variance, we construct 2 testing sets per task; testing set 1 is from June 2020 snapshot, and testing set 2 is from February 2022, all testing sets filtered the sequences that appeared in the training set. The details are listed in Table S7.

• Dataset 1: Enzyme and non-enzyme dataset

The training set in total has 469,134 records, 222,567 of which are enzymes and 246,567 are non-enzymes (Table S2). To make the data more inclusive, we did not filter any sequence in terms of length and homology, which is different from previous studies. An enzyme is labeled as 1 and a non-enzyme is labeled as 0.

• Dataset 2: Multifunctional enzyme dataset

The multifunctional enzyme dataset only contains enzyme data. The number of EC categories ranges from 1 to 8 (Table S3).

• Dataset 3: EC number dataset

Similar to the multifunctional enzyme dataset, the EC number dataset contains only enzyme records, 222,567 of which constitute the training dataset (covering 5,111 EC numbers). The test data include newly added EC numbers compared with the training data (Fig. S3), which means that these EC numbers do not appear in the training process, so predictive methods cannot handle this part of the EC numbers. Thus, we exclude the sequences with these EC numbers in the evaluation process.

### Proposed framework

**Fig. 6.**
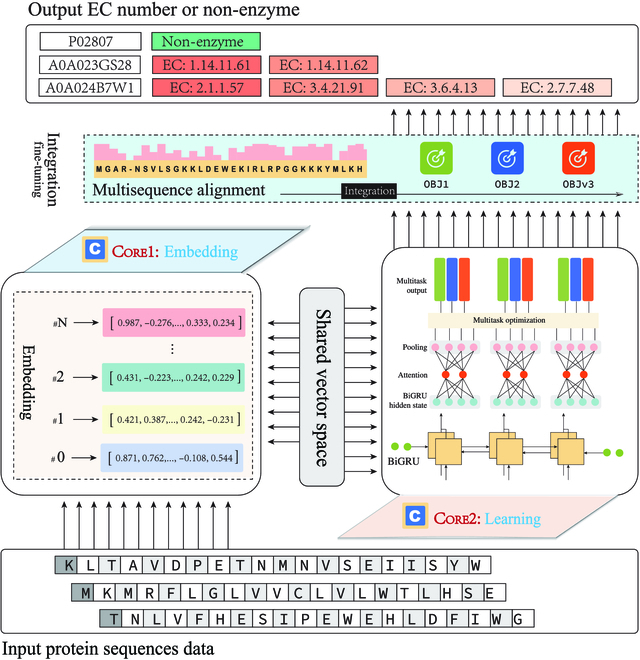
HDMLF is an explicitly designed dual-core driven framework for EC number prediction. It consists of 2 independent operation units—an embedding core and a learning core. The embedding core is tasked with converting protein sequences into features. The learning core is designed to address the specific biological tasks defined in the problem formulation section.

To develop a novel EC prediction method with cutting-edge performance, we proposed HDMLF, which is composed of an embedding core and a learning core. These 2 cores operate relatively independently. The embedding core is responsible for embedding protein sequences into a machine-readable matrix. The learning core is responsible for solving specific downstream biological tasks (e.g., enzyme and non-enzyme prediction, multifunctional enzyme prediction, and EC number prediction). The overall scheme of HDMLF is illustrated in Fig. 6.

• Core 1: Embedding

The objective of this core is to calculate the embedding representations for protein sequences. For protein sequence encoding/embedding, recent studies have shown the superior performance of deep learning-based methods compared to traditional methods [31,32]. Accordingly, we only compared one-hot encoding to show the difference between these 2 kinds of embedding in this study. Here, we adopted 3 different embedding methods to calculate the sequence embedding patterns that adequately represent protein sequences. The first one is the commonly used one-hot encoding [33]. The second is UniRep [22], an mLSTM “babbler” deep representation learner for proteins. We used the last layer for protein representation. The third is the ESM [23], a pretrained transformer language model for protein representation. We used representations from the 1st, 32nd, and 33rd layers as protein embeddings.

• Core 2: Learning

The learning core is specialized to perform specific biological tasks using a multitask learning (MTL) framework, which is implemented by a bidirectional GRU (BGRU) network with an attention mechanism. As shown in Fig. 6, the learning core uses embedding results as a unified input and uses BGRU to learn enzyme and non-enzyme prediction task (task 1), multifunction enzyme prediction task (task 2), and EC number prediction task (task 3) together. We use 3 multihead attention layers to learn and highlight interactive information among different tasks:

$$O_{h}=\text{softmax}\left(\frac{Q_{ht_{i}}K_{h}^{T}}{\sqrt{d_{h}}}\right)V_{h},i\in \left\{\text{task}\;1,\text{task}\;2,\text{task}\;3\right\}$$

where $\sqrt{d_{h}}$ is a scaling factor and *O_h_* is a one-head output of the attention layer, *Q_ht_i__* is the weight learning weight from task 1 to task 3, and $K_{h}^{T}$ represents the multitask learning hidden state vector for each learning layers.

The advantage of MTL is that multiple related learning tasks are solved simultaneously by exploiting commonalities and differences across relevant tasks [34], which considerably fits the current scenario. Compared with solving the 3 tasks separately or predicting EC number directly, MTL can improve the generalization performances of all the tasks because useful information contained in multiple related tasks is shared in the learning procedure. However, obtaining optimized weight parameters for all tasks will lead to a negative transfer problem that will hurt the learning performance [35]. To overcome this problem, here, we introduced a penalty parameter Ω to enforce a clustering of the task parameter vectors *a*_*t*1_, *a*_*t*2_, *a*_*t*3_ toward their mean that is controlled by a hyperparameter *λ*. Ω is defined as follows:

$$\Omega ={||\bar{a}||}^{2}+\sum_{t=1}^{t}‍\lambda_{t}||a_{t}-\bar{a}||$$

where $\bar{a}=\sum_{t=1}^{T}‍/T$ is the mean parameter vector, *T* represents the number of tasks; in this work, we set *λ*_1_ = 0.5, *λ*_2_ = 0.1, and *λ*_3_ = 0.4. The details of implementation and parameter settings can be found in the Supplementary Materials.

• Integration, fine-tuning, and output

As illustrated in Fig. 6, the final EC number prediction output is an integrated process. As shown in Eq. 4, we formulated this integrated process as an optimization problem:

$$\underset{F1}{\text{MAX}}\left\{f\left(\mathrm{ob}j_{1},\mathrm{ob}j_{2},\mathrm{ob}j_{3},\mathrm{sa}\right)\right\}$$ (4)

where *obj*_1_, *obj*_2_, and *obj*_3_ are the prediction results from task 1, task 2, and task 3, respectively, while *sa* is the predicted result from multiple sequence alignment. The integration and fine-tuning process aim to maximize the optimizing objective. In this work, the objective is the performance of prediction tasks in terms of the F1 score. We used a greedy strategy to perform this optimization.

### Compared baselines

To evaluate our proposed method comprehensively, we compared our proposed method with 4 existing state-of-the-art techniques with ‘GOOD’ usability (Supplementary Materials) and traditional sequence alignment method, which is provided by Diamond software. Four state-of-the-art techniques are CatFam, PRIAM (version 2), ECPred, and DeepEC.

### Evaluation metrics

To comprehensively evaluate the proposed method and existing baselines, we use 5 metrics to evaluate binary classification problems and 4 metrics to evaluate multiple classification problems. For the binary classification task, the evaluation criteria include ACC (accuracy), Precision, NPV (negative predictive value), Recall, and F1 value:

$$\text{ACC}=\frac{\text{TP}+\text{TN}}{\text{TP}+\text{FP}+\text{TN}+\text{FN}+\text{UP}+\text{UN}}$$ (5)

$$\text{Precision}=\frac{\text{TP}}{\text{TP}+\text{FP}}$$ (6)

$$\text{NPV}=\frac{\text{TN}}{\text{TN}+\text{FN}}$$ (7)

$$\text{Recall}=\frac{\text{TP}}{\text{TP}+\text{FN}+\text{UP}}$$ (8)

$$F1=\frac{2\times \text{Precision}\times \text{Recall}}{\text{Precision}+\text{Recall}}$$ (9)

where TP is the true-positive value, FP is the false-positive value, TN is the true-negative value, FN is the false-negative value, UP is unclassified-positive samples, and UN is unclassified-negative samples.

For multiple classification problems, the evaluation criteria included mACC (macro-average accuracy), mPR (macro-average precision), mRecall (macro-average recall), and mF1 (macro-average F1 value):

$$\text{mACC}=\frac{\sum_{i=1}^{n}‍{\text{ACC}}_{i}}{n},n=1,2,3,\cdots ,N$$ (10)

$$\text{mPR}=\frac{\sum_{i=1}^{n}‍{\text{PPV}}_{i}}{n},n=1,2,3,\cdots ,N$$ (11)

$$\text{mRecall}=\frac{\sum_{i=1}^{n}‍{\text{Recall}}_{i}}{n},n=1,2,3,\cdots ,N$$ (12)

$$\text{mF}1=\frac{2\times \text{mPR}\times \text{mRecall}}{\text{mPR}+\text{mRecall}}$$ (13)

where *N* represents the total number of classes, while ACC*_i_*, PPV*_i_*, and Recall*_i_* represent the accuracy, precision, and recall of the *i*th class in a one-versus-all mode [36], respectively.

### Web platform implementation

As shown in Fig. S8 the web platform uses Amazon ECR to store Docker images, which packages a set of bioinformatics software, such as Diamond and in-house Python scripts. We built a scalable, elastic, and easily maintainable batch engine using AWS Batch. This solution took care of dynamically scaling our computer resources in response to the number of runnable jobs in our job queue. Finally, we used AWS step functions to coordinate the components of our applications easily, process messages passed from AWS API Gateway, and invoke the workflows asynchronously. AWS API Gateway was used as the API server to handle the HTTP requests and route traffic to the correct backends. The static website was hosted by AWS S3 and sped up using AWS CloudFront.

## Data Availability

The code of HDMLF, the training data, and the prediction results are available at https://github.com/kingstdio/ECRECer https://github.com/tibbdc/ECRECer.
