# Supplementary material for: Enzyme Commission Number Prediction and Benchmarking with Hierarchical Dual-core Multitask Learning Framework
Source: Research (Wash D C). 2023 May 31;6:0153. doi: 10.34133/research.0153 (PMC10232324; doi:10.34133/research.0153)
Supplement: Supplementary 1 — Tables S1 to S13 Figs. S1 to S8 [file research.0153.f1.pdf]

★ Supplemental Document

ECRECer: Enzyme Commission Number Prediction and Benchmarking with Hierarchy Dual-core Multitask Learning Framework

Zhenkun Shi[1, 2], Rui Deng [3], Qianqian Yuan[1, 2], Zhitao Mao[1, 2], Ruoyu Wang[1, 2], Haoran Li[1, 2], Xiaoping Liao[1, 2, (✉)], Hongwu Ma[1, 2, (✉)]

[1]Biodesign Center, Key Laboratory of Engineering Biology for Low-carbon Manufacturing, Tianjin Institute of Industrial Biotechnology, Chinese Academy of Sciences, 300308, Tianjin, China.
[2]National Technology Innovation Center of Synthetic Biology, 300308, Tianjin, China
[2]College of Biotechnology, Tianjin University of Science & Technology, Tianjin, China.

✉{liao_xp, ma_hw}@tib.cas.cn

Abstract

This is a supplementary document for the paper "ECRECer: Enzyme Commission Number Prediction and Benchmarking with Hierarchy Dual-core Multitask Learning Framework". It provides details on preparing the data, selecting models, fine-tuning parameters, performance evaluation, as well as supplementary figures and tables that provide experimental details and support our conclusions. It also includes information on how to use the web service and offline bundles for high-throughput EC number prediction.

1 SI Related Work

As EC number prediction is at the core of enzyme functional annotation, a large number of relevant computational techniques have been developed to assign EC numbers to unknown protein sequences. In this section, we will introduce seven of the most representative ones, ordered by their time of publication. The seven representative tools are listed in Table 1. Next, we evaluated these tools based on the latest update time, distribution type (standalone packages, online web-service, or both), usability ('YES' if it is available for use, 'NO' if it is not available, 'Good' if it can be used for high-throughput prediction) and citations of these tools up to 26 Aug 2021.

1.1 CatFam

CatFam [10] is a profile-controlled, sequence-based database that can be used to infer the catalytic functions of proteins. CatFam uses an adjustable false positive rate to generate databases on-demand for different needs, such as functional annotation with different precision and hypothesis generation with moderate precision but better recall. CatFam uses profile-specific thresholds to ensure equal precision for each profile and

Table 1. Usability of 7 EC prediction tools

Tools	Last update	Type	Usability	Citations
CatFam[1]	2009	standalone	GOOD	71
SVMProt[2]	2016	online	NO	88
PRIAM_V2[3]	2018	both	GOOD	365
DEEPre[4]	2018	online	YES	132
ECPred[5]	2018	both	GOOD	40
DEEPEC[6]	2019	standalone	GOOD	49
BENZ WS[7]	2021	online	YES	0

1. http://www.bhsai.org/downloads/catfam.tar.gz
2. http://bidd.group/cgi-bin/svmprot/svmprot.cgi
3. http://priam.prabi.fr/REL_JAN18/index_jan18.html
4. http://www.cbrc.kaust.edu.sa/DEEPre/index.html
5. https://ecpred.kansil.org/
6. https://bitbucket.org/kaistsystemsbiology/deepec/src/master/
7. https://benzdb.biocomp.unibo.it/

ensure the best performance for all tasks. Comparison experiments were conducted based on three test sets and 13 bacterial genomes. The results demonstrated that CatFam outperforms PRIAM in terms of precision and coverage. CatFam has been developed for more than 12 years. Although the precision is not as good as in the latest ones, the recall remains good, and its code is still available. We, therefore, used CatFam as one of our baselines in this work.

1.2 SVM-Prot

SVM-Prot V2016 [6] is a machine-learning method that was first published in 2003 and then updated in 2016. SVM-Prot is supplementary for predicting diverse classes of proteins compared with distantly-related or homologous-related methods. SVM-Prot employs 13 manually curated physicochemical features of proteins as inputs, nine of which are from Pse-in-One [7], while the remaining four are self-calculated, such as molecular weight and solubility. The algorithm then uses these features to train an integrated SVM, KNN, PNN, and Blast model, to predict the EC numbers for new proteins. Sensitivity, precision, and specificity are evaluated on an independent evaluation dataset, which demonstrated the outstanding performance of SVM-Prot. However, to train an SVM classifier the time complexity is $O(n^2p+n^3)$ [1], which is extremely time-consuming. More importantly, the web service provided by SVM-Prot is no longer available, and they did not provide their code for reimplementation and evaluation. Hence, the usability of SVM-Prot is weak.

1.3 PRIAM-V2

PRIAM V2 [3] s a rules-based method for automated enzyme annotation with EC numbers proposed in 2003, with an updated version V2 published in 2018. It takes protein or nucleotide sequences as inputs and annotates them with EC numbers on an individual sequence level or a genome level. PRIAM utilizes a set of signatures composed of position-specific scoring matrices and patterns for sequence embedding, which is tailored for each enzyme entry to build its model. PRIAM uses the whole Swiss-Prot database to learn parameters and evaluate the method as well. The advantage of PRIAM is its high recall, and the code is available. Accordingly, we used PRIAM V2 as one of our baselines.

1.4 DEEPre

DEEPre [5] is a supervised end-to-end feature selection and classification model that uses a convolutional neural network (CNN) with a level-by-level strategy to predict enzyme functions. Unlike the above-mentioned method that needs manually curated features, DEEPre takes the raw sequence encoding as inputs, then extracts convolutional and sequential features from the raw encoding based on the classification result to directly boost the model performance. DEEPre is good at determining the main classes of enzymes on a separate low-homology dataset, while the performance is suboptimal when determining the fourth level EC numbers. DEEPre provides a webserver for the public but does not provide the source code for reimplementation and evaluation, and the webserver is not capable of high-throughput prediction. Thus, this algorithm is usable but not user-friendly.

1.5 ECPred

ECPred [4] is a supervised hierarchical enzyme function prediction tool based on an ensemble of machine learning that can predict EC numbers to the fourth level. ECPred trains an independent model for each EC number level and uses three predictors, called SPMap, BLAST-kNN, and Pepstats-SVM, to integrate the output. ECPred was trained and validated using the enzyme entries located in the Swiss-Prot database. ECPred ingeniously constructed a positive set and a negative set to finely control the prediction performance. The experimental results showed its outstanding performance at level 0 EC number prediction. ECPred was published in late 2018. The most significant point of ECPred is its user-friendly workflow that provides both a web service, standalone packages, and the source code. Accordingly, we used ECPred as one of our baselines in this work.

1.6 DEEPEC

DeepEC [8] is a deep learning method that enables high-quality and high-throughput prediction of EC numbers. DeepEC uses three CNN as its major engine and homology analysis as its supplementary engine to conduct EC number prediction. DeepEC predicts if the given amino sequence is an enzyme in the first CNN layer, and then specifies the third level of EC numbers in the second CNN layer, after which it assigns the fourth level in the final CNN layer. The primary objective of DeepEC is high precision, low computing time, and low disk space requirements. DeepEC is sensitive in detecting the effects of mutated domains/binding site residues. DeepEC did not provide a source code for self-training and reimplementation. It only provides well-trained parameters for local installation and prediction. However, no webserver is given. Considering its good performance in precision, we also used use DeepEC as one of our baselines in this work.

1.7 BENZ WS

BENZ WS [2] is the latest published web service for four-level EC number annotation. It was first published in May 2021. BENZ WS filters a target sequence with a combined system of HMMs and PFAMs, after which it returns an associated four-level EC number if successful. BENZ WS can annotate both mono- and multifunctional enzymes. Compared with DEEPre and ECPred, BENZ WS is superior in terms of the true positive rate. However, the performance of BENZ WS is relatively inferior in terms of the false-negative rate. BENZ WS only provides a web interface to the end-user, so usability is given, but no source code or standalone suite is available, and the

computational time is long. We therefore did not use BENZ WS as a baseline in this work.

2 SI Appendix Materials and Methods

2.1 Preprocessing

There are six steps (s1-s6) in data preprocessing:

- s1: remove the records with identical IDs, but changed sequences (updated sequences);
- s2: for duplicated records, only keep one;
- s3: make the EC numbers uniform and remove unnecessary spaces;
- s4: based on the EC number, assign a unique label for each sequence;
- s5: organize a uniform dictionary for EC label mapping;
- s6: add enzyme catalytic function quantity labels to protein sequences.

2.2 Dataset

A commonly used EC number prediction dataset is the EzyPred dataset from Shen and Zhou, published in 2007 [9]. The EzyPred dataset is a two-level EC number dataset that was extracted from the ENZYME database (released May 1, 2007), with a 40% sequence similarity cutoff. This dataset contains 9,832 two-level specified enzymes and 9850 non-enzymes. The details of this dataset can be found in their published paper [9]. This dataset can only be used to predict two-level EC numbers, and the volume of this dataset is unsuitable for machine learning. Accordingly, the majority of the later studies used a similar approach to extract and construct datasets from Swiss-Prot [8, 5]. The typical steps of constructing the dataset are as follows:

1) Obtain the latest reviewed protein data from Swiss-Prot and label the sequences as enzyme or none-enzyme utilizing the protein annotation.
2) Exclude the multifunctional enzymes and those enzymes with incomplete EC number annotations.
3) Exclude enzymes by sequence length, a typical threshold is $length \in [50, 50000]$.
4) Use homology analysis tools to remove redundant sequences. The similarity threshold is manually determined, and a typical threshold is 40%.
5) Randomly rearrange filtered enzyme data and randomly pick non-enzyme data with a similar size, then mix these data together as a standard dataset.
6) Split the standard dataset into a training set and a testing set using a typical 8:2 ratio or split the standard dataset into a training set, validation set, and testing set using a typical 7:1:2 ratio.

However, these principles of dataset construction were explicitly designed for the EC number prediction of monofunctional enzymes and are not suitable for multifunctional enzymes. Moreover, the construction of training and testing datasets using randomly mixed data is not in accordance with the facts and may lead to information leaks. Beyond that, filtering sequences by length and homology may obscure patterns and other information, which will reduce the learning performance. Therefore, the steps of constructing the dataset in this work were more straightforward:

1*) Obtain the latest reviewed protein data from Swiss-Prot and label the sequences with three label vectors: enzyme or none-enzyme, monofunctional (labeled 1 or 0) or multifunctional enzyme (labeled with function counts), EC number (monofunctional enzymes have a single EC number, multifunctional enzymes have more than one EC number).

2*) Rearrange the protein sequence order by annotation updated date.

3*) Use the latest four-year data as the testing set, while the rest is the training and validation set.

4*) We constructed two test sets to validate the methods' prediction efficiency over time. The first one is built using Sprot data from February 2018 to June 2020, namely testset_20, and the second one is from February 2018 to February 2022, namely testset_22.

The implementation can be seen in chapter 5 and by referring to our source codes.

2.2.1 Task 1 Enzyme and Non-enzyme Dataset

Based on the above mentioned three principles, the enzyme and non-enzyme dataset (Table 2) uses the latest 3 years of Swiss-Prot data as the testing set, and the data before as the training set.

Table 2. Description of the Enzyme and Non-enzyme Dataset

ITEM	Training set	testset_20	testset_22
Enzyme	222,567	3,304	5,111
Non-enzyme	246,567	3,797	5,503
Total	469,134	7,101	10,614

2.2.2 Task 2 Multifunctional Enzyme Dataset

For the multifunctional enzyme prediction dataset, to minimize distractions from non-enzymes and balance the dataset, we excluded the non-enzyme data (Table 3). The remaining enzyme data were labeled based on the number of functions (i.e., 1, 2, ...,8). The details are listed below:

Table 3. Description of Multifunctional Enzyme Dataset

Functions	Records			Functions	Records		
	Train	Test1	Test2		Train	Test1	Test2
1	210,788	3,273	4,656	5	206	6	7
2	9,943	208	337	6	80	2	10
3	993	60	84	7	27	1	1
4	525	7	13	8	5	0	3

Train: Training set
Test1: Testset_20
Test2: Testset_22

2.2.3 Task 3 Enzyme Commission Number Dataset

Following the three-datasets construction principle, the enzyme commission (EC) number dataset filtered the non-enzyme data after preprocessing. For a comprehensive and fair comparison with the state-of-the-art method DeepEC, we set the end-time of

the training dataset to February 2018. This is because DeepEC only collected data before February 2018 for model training. If we use more recent data it will lead to an information leak problem. The dataset (Table 4) details are listed below:

Table 4. Description of the Enzyme Commission Number Dataset

Item	Trainingset	testset_20	testset_22
Monofunctional	210,788	3,052	4,656
Multifunctional	11,779	252	455
Distinct EC numbers	4,854	937	1,355
Incomplete EC numbers	209	128	137
Complete EC numbers	4,645	809	1,218
Oxidoreductases	34,169	1,065	1,615
Transferases	79,570	1,009	1,567
Hydrolases	56,749	676	989
Lyases	20,747	346	608
Isomerases	11,927	108	184
Ligases	25,254	156	244
Translocases	-	57	83
Set size	222,567	7,101	5,111

3 Models

3.1 Optimized parameters

```
# Bi-GRU and Attention optimized parameters

Layer (type)                    Output Shape       Param #
==========================================================
input (InputLayer)              n*(1, 1280)        0
bi-gru (Bidirectional)          (1, 256)           1082880
attention (Attention)           (256)              8225
dense1 (Dense)                  (64)               16448
lniear (Dense)                  (num_of(Y_label))  130
==========================================================
Total params: 1,107,683
Trainable params: 1,107,683
Non-trainable params: 0
```

3.2 Integration, fine-tuning, and production

As illustrated in Fig. 1, the final EC number prediction output is an integrated process. As shown in SE. 1, we formulated this integrated process as an optimization problem:

$$\underset{F1}{MAX}\{f(obj_1, obj_2, obj_3, sa)\} \tag{SE.1}$$

where ag_1, ag_2, and ag_3 are the predicted results from DMLF finetued for task1, task2, task3, respectively, while sa is the predicted results from multiple sequence alignment. The integration and fine-tuning process aim to maximize the optimizing objective. In

this work, the objective was the performance of EC number prediction in terms of the F1 score. We used a greedy strategy to finish this optimization.

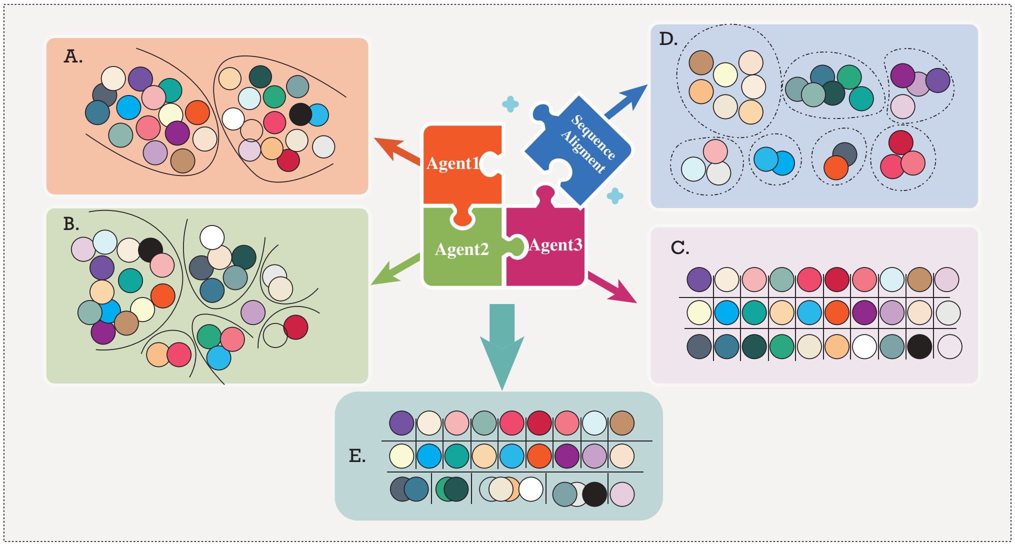

Figure 1. The integration and fine-tuning process before output.

4 SI Appendix Figures

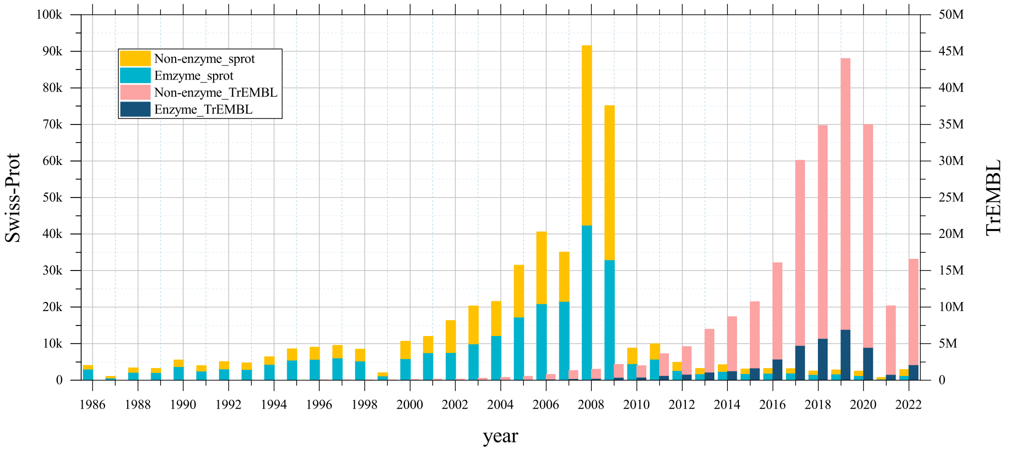

Figure 2. The number of records integrated into TrEMBL vs. Swiss-Prot since 1986.

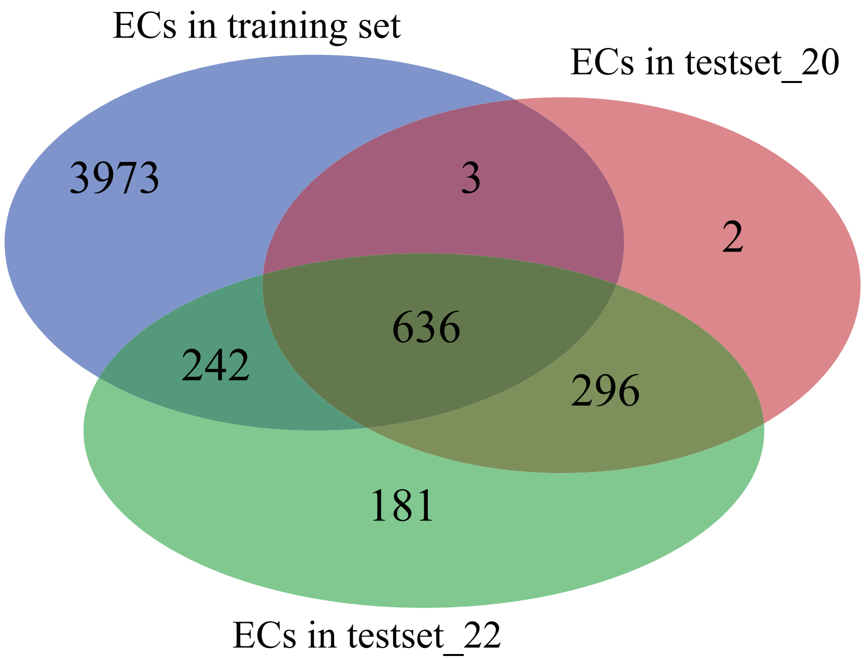

Figure 3. Venn diagram of the training and testing datasets

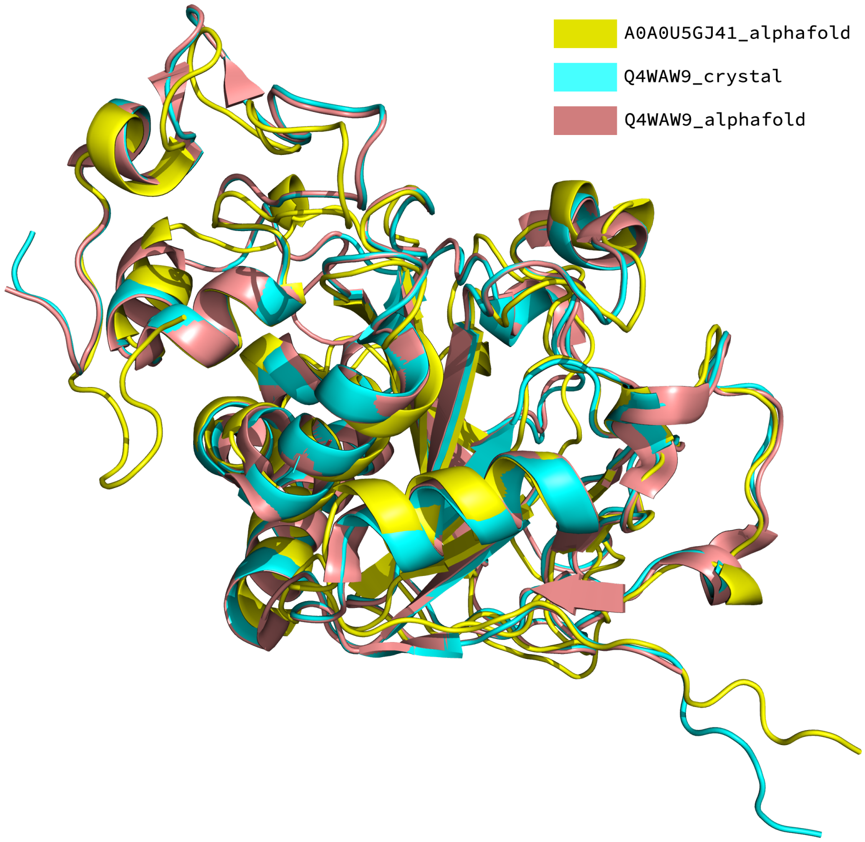

Figure 4. Structure alignment of proteins A0A0U5GJ41 and Q4WAW9.

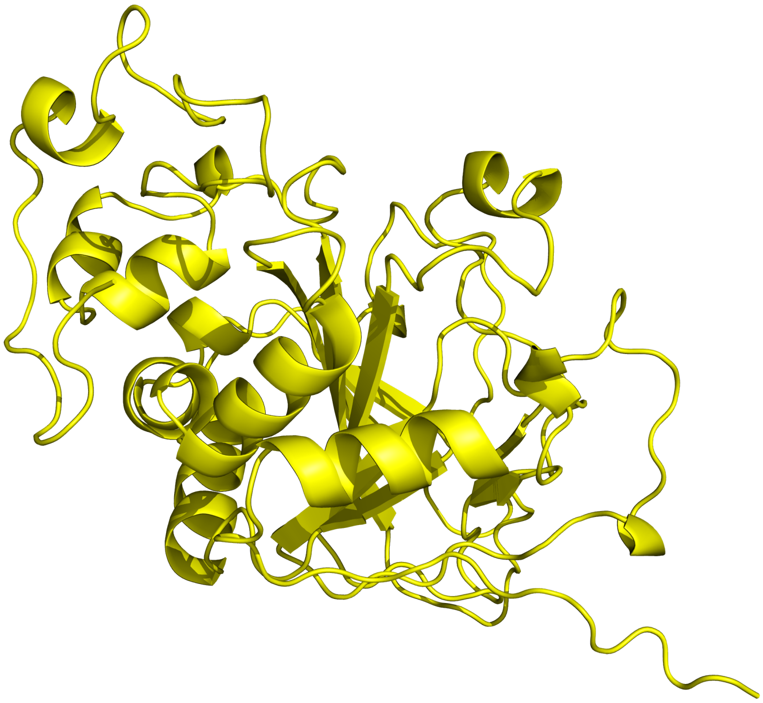

Figure 5. Structure of protein A0A0U5GJ41 (predicted using alphfold2).

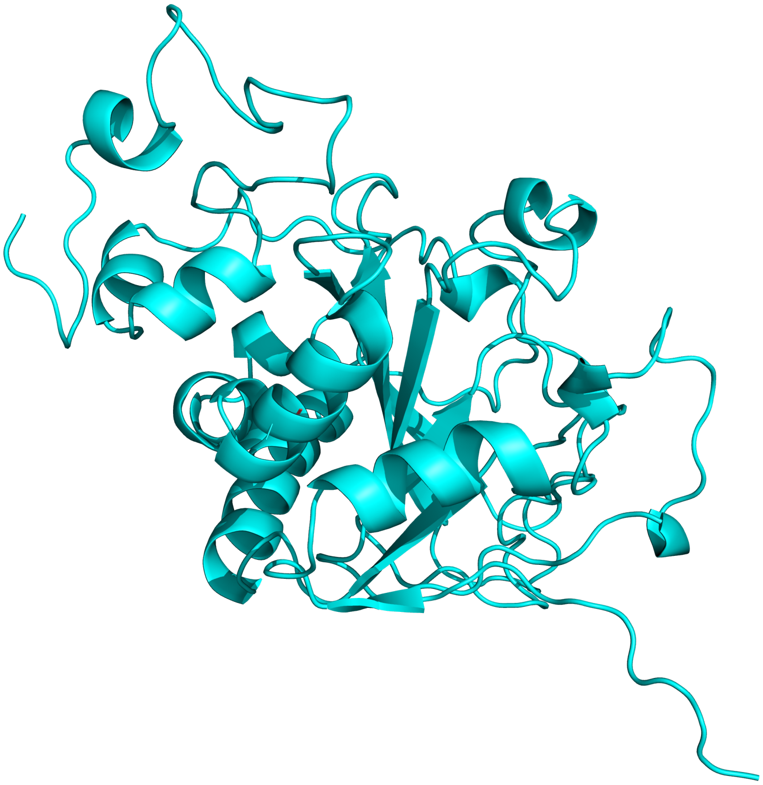

Figure 6. Structure of protein Q4WAW9 (predicted using alphfold2).

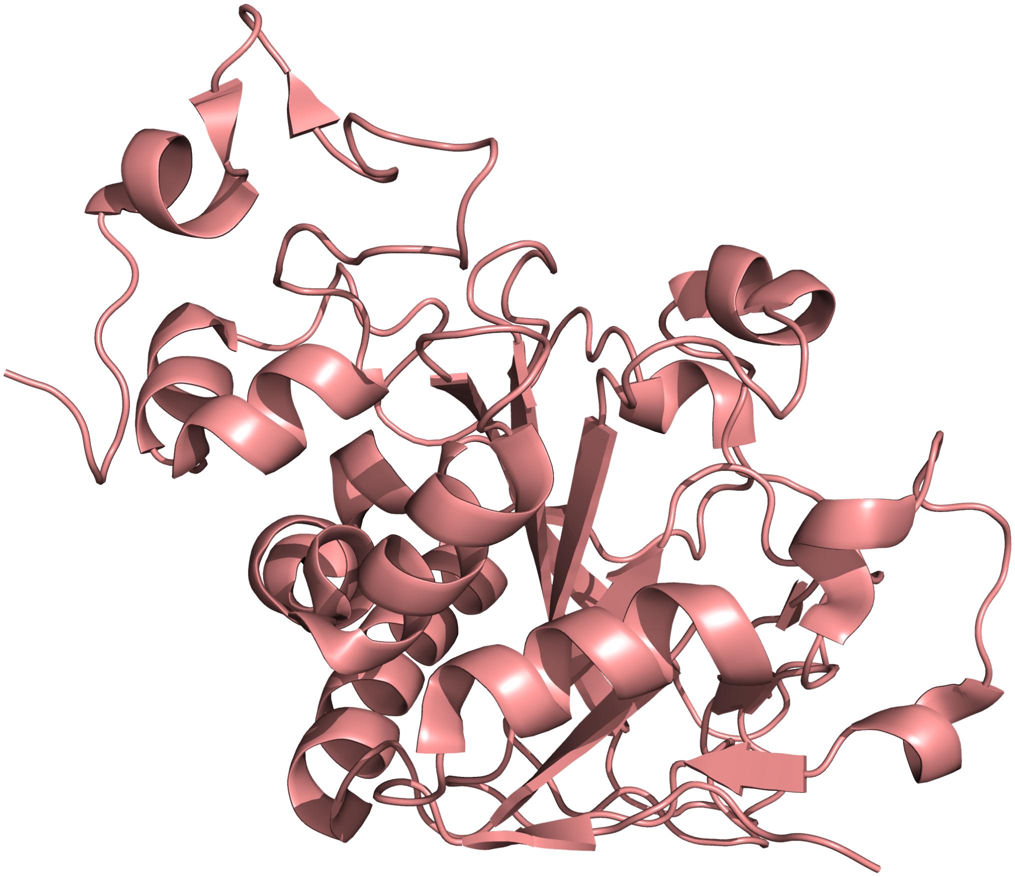

Figure 7. Structure for protein Q4WAW9 (alphfold2 predicted).

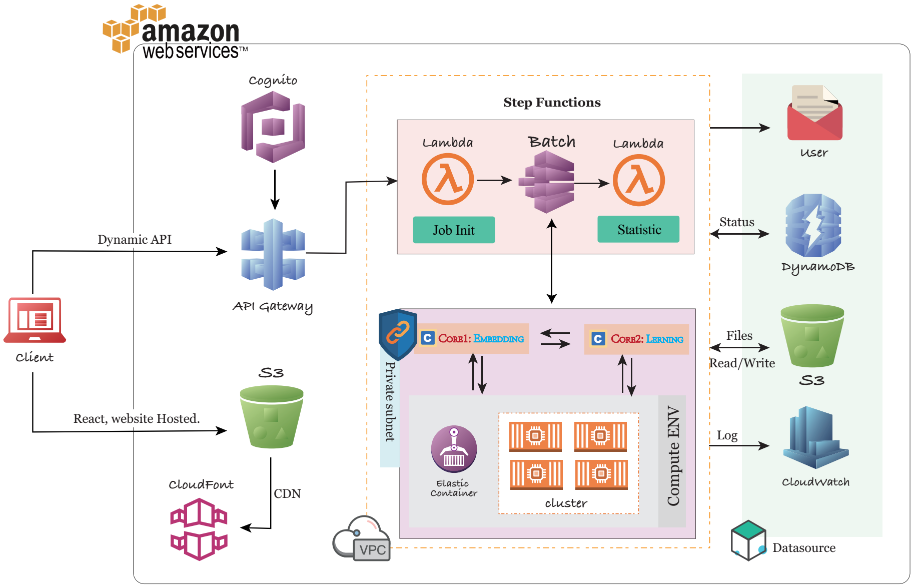

Figure 8. The software architecture of the web platform.

5 SI Algorithm

Algorithm 1 Prepare benchmarking datasets

```
download raw data from uniprot.org                 ▷ prepare_task_dataset.ipynb # Step 3
    train_data ← uniprot_sprot − only2018_02.tar.gz
    test_data ← uniprot_sprot − only2020_06.tar.gz
    test_data ← uniprot_sprot − only2022_02.tar.gz
extract protein records from downloaded data       ▷ prepare_task_dataset.ipynb # Step 4
    extract protein id
    extract protein name
    extract protein ec_number
    extract protein sequence as seq
    format ec_number and seq
    caculate protein arrtruibutes.                 ▷ exact_ec_from_uniprot.py
preprocessing protein records                      ▷ prepare_task_dataset.ipynb # Step 6
    drop duplicates by seq
    remove changed seq with same id
    format ec_number in standard four level like: -.-.-.-
    trim ec_number and seq strings
get esm embedding                                  ▷ prepare_task_dataset.ipynb # Step 6.6
get unirep embeeding                               ▷ prepare_task_dataset.ipynb # Step 6.7
Construct task1 dataset ds1                        ▷ prepare_task_dataset.ipynb # Step 7.1
Construct task2 dataset ds2                        ▷ prepare_task_dataset.ipynb # Step 7.2
Construct task3 dataset ds3                        ▷ prepare_task_dataset.ipynb # Step 7.3
```

Algorithm 2 EC Number Prediction

```
load trainset and testset from ds3                 ▷ task3.ipynb # Step 2
load embedding features                            ▷ task3.ipynb # Step 3
conduct sequence alignment                         ▷ task3.ipynb # Step 4
transfer EC number to model labels                 ▷ task3.ipynb # Step 5
train EC prediction model                          ▷ task3.ipynb # Step 6
do EC prediction                                   ▷ task3.ipynb # Step 7
return prediction results
```

Algorithm 3 Enzyme or Non-enzyme Prediction

```
load trainset and testset from ds1                          ▷ task1.ipynb # Step 2
conduct sequence alignment                                  ▷ task1.ipynb # Step 3
embedding comparison                                        ▷ task1.ipynb # Step 4
    one-hot embedding                                       ▷ task1.ipynb # Step 4.1
    unirep embedding                                        ▷ task1.ipynb # Step 4.2
    esm layer 33 embedding                                  ▷ task1.ipynb # Step 4.3
    esm layer 32 embedding                                  ▷ task1.ipynb # Step 4.4
    esm layer 0 embedding                                   ▷ task1.ipynb # Step 4.5
DMLF for enzyme or non-enzyme prediction                    ▷ task1.ipynb # Step 5
    learn model using KNN method on train_data
    predict enzyme or non-enzyme on test_data using learned model
    integrate KNN prediction with sequence alignment prediction
if sequence alignment found homologous sequence then
    use alignment results as prediction
else
    use KNN results as prediction
return prediction
```

Algorithm 4 Enzyme Catalytic Function Quantity Prediction

```
load trainset and testset from ds2                          ▷ task2.ipynb # Step 2
load esm32 embedding features                               ▷ task2.ipynb # Step 3
conduct single or multi functions prediction benchmarking sp ▷ task2.ipynb # Step
4.1
conduct 2-8 functions prediction benchmarking mp            ▷ task2.ipynb # Step 4.2
do sequences alignment
do function counts prediction
integrate and output results                                ▷ task2.ipynb # Step 5.3
if sequence alignment found homologous sequence then
    use alignment results as prediction
else if sp prediction is single functional then
    prediction is sp results
else
    use mp results
return prediction results
```

6 SI Appendix Tables

Table 5. Benchmarking Data Description

ITEM	Snapshot		
	February-2018	June-2020	February-2022
Records	556,825	563,972	567,483
Duplicate Removal	469,129	476,006	479,426
Non-enzyme	246,562	247,319	247,338
Enzyme	222,565	228,687	232,088
Distinct EC	4854	5306	5570

Table 6. Protein Sequences Embedding Performance Comparation on Enzyme or Non-Enzyme Prediction Task

Method	Baseline	ACC	PPV	NPV	RC	F1	Confusion Matrix			
							TP	FP	FN	TN
Logistic Regression	one-hot	0.6473	0.5886	0.7120	0.6924	0.6363	2478	1732	1101	2722
	Unirep	0.8368	0.8593	0.8222	0.7578	0.8053	2712	444	867	4010
	ESM0	0.7561	0.7209	0.7857	0.7385	0.7296	2643	1023	936	3431
	ESM32	0.9066	0.9209	0.8964	0.8648	0.8919	3095	266	484	4188
	ESM33	0.9032	0.9204	0.8909	0.8567	0.8874	3066	265	513	4189
KNN	one-hot	0.6330	0.6686	0.6222	0.3495	0.4591	1251	620	2328	3834
	Unirep	0.8486	0.8670	0.8363	0.7798	0.8211	2791	428	788	4026
	ESM0	0.8246	0.7892	0.8556	0.8273	0.8078	2961	791	618	3663
	ESM32	0.9294	0.9411	0.9208	0.8977	0.9189	3213	201	366	4253
	ESM33	0.9273	0.9360	0.9208	0.8983	0.9167	3215	220	364	4234
XGboost	one-hot	0.7087	0.6851	0.7256	0.6407	0.6621	2293	1054	1286	3400
	Unirep	0.8651	0.8885	0.8494	0.7972	0.8404	2853	358	726	4096
	ESM0	0.8282	0.8197	0.8346	0.7877	0.8034	2819	620	760	3834
	ESM32	0.9254	0.9540	0.9057	0.8748	0.9127	3131	151	448	4303
	ESM33	0.9157	0.9443	0.8962	0.8617	0.9011	3084	182	495	4272
Decision tree	one-hot	0.6283	0.5889	0.6562	0.5488	0.5681	1964	1371	1615	3083
	Unirep	0.7966	0.7951	0.7976	0.7320	0.7623	2620	675	959	3779
	ESM0	0.7621	0.7437	0.7758	0.7111	0.7270	2545	877	1034	3577
	ESM32	0.8422	0.8550	0.8334	0.7776	0.8145	2783	472	796	3982
	ESM33	0.8311	0.8442	0.8223	0.7614	0.8006	2725	503	854	3951
Random forest	one-hot	0.7162	0.6768	0.7493	0.6946	0.6856	2486	1187	1093	3267
	Unirep	0.8634	0.9151	0.8328	0.7645	0.8330	2736	254	843	4200
	ESM0	0.8539	0.8636	0.8470	0.7980	0.8295	2856	451	723	4003
	ESM32	0.9157	0.9657	0.8841	0.8407	0.8989	3009	107	570	4347
	ESM33	0.9161	0.9610	0.8871	0.8460	0.8999	3028	123	551	4331
GBDT	one-hot	0.6775	0.6163	0.7461	0.7315	0.6690	2618	1630	961	2824
	Unirep	0.8332	0.8738	0.8091	0.7312	0.7962	2617	378	962	4076
	ESM0	0.8210	0.8100	0.8293	0.7815	0.7955	2797	656	782	3798
	ESM32	0.8720	0.9050	0.8507	0.7963	0.8472	2850	299	729	4155
	ESM33	0.8658	0.9017	0.8431	0.7843	0.8389	2807	306	772	4148

Table 7. Protein Sequence Embedding Performance on Multifunctional Enzyme Prediction Task

Baseline	Method	ACC	Precision-Macro	Recall-Macro	F1-Macro
Logistic regression	One-hot	0.9016	0.4485	0.2133	0.2206
	Unirep	0.9234	0.8462	0.1428	0.1372
	ESM0	0.9237	0.9891	0.1429	0.1372
	ESM32	0.9168	0.8205	0.3100	0.3719
	ESM33	0.9210	0.7792	0.4365	0.4897
KNN	One-hot	0.9180	0.6498	0.3601	0.3511
	Unirep	0.9044	0.5790	0.1479	0.1474
	ESM0	0.9156	0.6195	0.4261	0.4672
	ESM32	0.9274	0.6317	0.5459	0.5773
	ESM33	0.9280	0.7974	0.5644	0.5994
XGboost	One-hot	0.9252	0.8941	0.2374	0.2841
	Unirep	0.9192	0.8822	0.1480	0.1475
	ESM0	0.9258	0.8512	0.3878	0.4332
	ESM32	0.9389	0.9422	0.5101	0.5931
	ESM33	0.9380	0.9441	0.4626	0.5405
Decision tree	one-hot	0.8593	0.3079	0.2185	0.2305
	Unirep	0.8647	0.5951	0.1430	0.1440
	ESM0	0.8786	0.5263	0.2531	0.2869
	ESM32	0.8874	0.3937	0.5412	0.3984
	ESM33	0.8814	0.3948	0.3862	0.2604
Random forest	One-hot	0.9262	0.9419	0.2397	0.2887
	Unirep	0.9210	0.8462	0.1424	0.1370
	ESM0	0.9280	0.9421	0.3869	0.4317
	ESM32	0.9343	0.9394	0.4640	0.5398
	ESM33	0.9322	0.9271	0.4283	0.4997
GBDT	One-hot	0.9125	0.1820	0.3125	0.1680
	Unirep	0.9228	0.8462	0.1427	0.1371
	ESM0	0.9240	0.5991	0.4407	0.3403
	ESM32	0.9271	0.6479	0.3135	0.3643
	ESM33	0.9231	0.6347	0.4828	0.3178

Table 8. EC Number Prediction Performance Comparation

basline	mACC	mPR	mRecall	mF1	dataset
ECPred	0.2458	0.8042	0.2630	0.2955	testset_20
DeepEC	0.3011	0.8121	0.3794	0.3011	
CatFam	0.2760	0.8323	0.3507	0.2760	
PRIAM-V2	0.2457	0.2080	0.7848	0.2457	
Ours	0.8619	0.6900	0.8388	0.6176	
ECPred	0.2350	0.8552	0.1620	0.1820	testset_22
DeepEC	0.2003	0.7910	0.2879	0.1049	
CatFam	0.2763	0.8858	0.1916	0.0837	
PRIAM-V2	0.2426	0.2503	0.7526	0.0452	
Ours	0.8742	0.7721	0.8090	0.6445	

Table 9. Enzyme or None-enzyme Prediction Performance Comparation-testset_20

Baseline	ACC	PPV	NPV	RC	F1	Confusion Matrix					
						TP	FP	FN	TN	UP	UN
testset_20											
ECPred	0.7219	0.8218	0.9190	0.8463	0.8339	3029	657	244	277	306	1027
DeepEC	0.6715	0.9468	0.6300	0.2783	0.4301	996	56	2583	4398	0	0
CatFam	0.6502	0.8050	0.6214	0.2836	0.4194	1015	246	2564	4208	0	0
PRIAM$V2$	0.7410	0.6486	0.8967	0.9137	0.7586	3270	1772	309	2682	0	0
Ours	0.9312	0.9525	0.9160	0.8899	0.9201	3185	159	394	4295	0	0
testset_22											
ECPred	0.8021	0.7525	0.8655	0.8775	0.8102	4485	1454	144	4028	21	482
DeepEC	0.6383	0.9441	0.5906	0.2645	0.4133	1352	80	3759	5423	0	0
CatFam	0.5944	0.9278	0.5619	0.1710	0.2888	874	68	4237	5435	0	0
PRIAM-V2	0.7473	0.6780	0.8721	0.9051	0.7753	4626	2197	85	3306	0	0
Ours	0.9324	0.9179	0.9493	0.9549	0.9361	5255	470	248	4641	0	0

Table 10. Multifunctional Enzyme Prediction Performance Comparation

basline	accuracy	precision-macro	recall-macro	f1-macro
testset_20				
ECPred	0.9189	0.8654	0.2493	0.1197
DeepEC	0.2216	0.1238	0.0330	0.0522
CatFam	0.9237	0.9891	-	-
PRIAM-V2	0.1311	0.2292	0.0446	0.0747
Ours	0.9171	0.5837	0.5520	0.5605
testset_22				
ECPred	0.9069	0.8790	0.2217	0.1057
DeepEC	0.9063	0.8177	0.1354	0.1389
CatFam	0.9102	0.6622	0.1592	0.1749
PRIAM-V2	0.1327	0.0095	0.9309	0.0032
Ours	0.9245	0.7068	0.5956	0.5745

Table 11. First-level EC Prediction Performance Comparation

baslineName	accuracy	precision-macro	recall-macro	f1-macro
testset_20				
ECPred	0.7879	0.9127	0.2824	0.3193
DeepEC	0.3197	0.8513	0.4247	0.3994
CatFam	0.3558	0.8579	0.3684	0.3287
PRIAM-V2	0.3037	0.2996	0.8969	0.3052
Ours	0.9125	0.7679	0.8571	0.6365
testset_22				
ECPred	0.7803	0.9144	0.2200	0.1883
DeepEC	0.2411	0.8628	0.4524	0.4096
CatFam	0.3571	0.9172	0.2162	0.1253
PRIAM-V2	0.3039	0.2960	0.9003	0.4045
Ours	0.9130	0.7815	0.8317	0.6663

Table 12. Second-level EC Prediction Performance Comparation

baslineName	accuracy	precision-macro	recall-macro	f1-macro
testset_20				
ECPred	0.5479	0.8389	0.2725	0.3135
DeepEC	0.3153	0.8510	0.4054	0.3409
CatFam	0.3550	0.8430	0.3611	0.3091
PRIAM-V2	0.2882	0.2895	0.8623	0.2815
Ours	0.8966	0.7654	0.8425	0.6253
testset_22				
ECPred	0.5572	0.9029	0.2132	0.1878
DeepEC	0.2275	0.8625	0.3154	0.3717
CatFam	0.3390	0.9133	0.2016	0.1184
PRIAM-V2	0.2882	0.2790	0.8630	0.2903
Ours	0.8865	0.7801	0.8246	0.6605

Table 13. Third-level EC Prediction Performance Comparation

baslineName	accuracy	precision-macro	recall-macro	f1-macro
testset_20				
ECPred	0.2458	0.8042	0.2630	0.2955
DeepEC	0.3011	0.8121	0.3794	0.3011
CatFam	0.2760	0.8323	0.3507	0.2760
PRIAM-V2	0.2457	0.2080	0.7848	0.2457
Ours	0.8619	0.6900	0.8388	0.6176
testset_22				
ECPred	0.2350	0.8552	0.1620	0.1820
DeepEC	0.2003	0.7910	0.2879	0.1049
CatFam	0.2763	0.8858	0.1916	0.0837
PRIAM-V2	0.2426	0.2503	0.7526	0.0452
Ours	0.8742	0.7721	0.8090	0.6445

References

1. A. Abdiansah and R. Wardoyo. Time complexity analysis of support vector machines (svm) in libsvm. *International journal computer and application*, 128(3):28–34, 2015.

2. D. Baldazzi, C. Savojardo, P. L. Martelli, and R. Casadio. Benz ws: the bologna enzyme web server for four-level ec number annotation. *Nucleic Acids Research*, 49:w60–w66, 2021.

3. C. Claudel-Renard, C. Chevalet, T. Faraut, and D. Kahn. Enzyme-specific profiles for genome annotation: Priam. *Nucleic acids research*, 31(22):6633–6639, 2003.

4. A. Dalkiran, A. S. Rifaioglu, M. J. Martin, R. Cetin-Atalay, V. Atalay, and T. Doğan. Ecpred: a tool for the prediction of the enzymatic functions of protein sequences based on the ec nomenclature. *BMC bioinformatics*, 19(1):1–13, 2018.

5. Y. Li, S. Wang, R. Umarov, B. Xie, M. Fan, L. Li, and X. Gao. Deepre: sequence-based enzyme ec number prediction by deep learning. *Bioinformatics*, 34(5):760–769, 2018.

6. Y. H. Li, J. Y. Xu, L. Tao, X. F. Li, S. Li, X. Zeng, S. Y. Chen, P. Zhang, C. Qin, C. Zhang, et al. Svm-prot 2016: a web-server for machine learning prediction of protein functional families from sequence irrespective of similarity. *PloS one*, 11(8):e0155290, 2016.

7. B. Liu, F. Liu, X. Wang, J. Chen, L. Fang, and K.-C. Chou. Pse-in-one: a web server for generating various modes of pseudo components of dna, rna, and protein sequences. *Nucleic acids research*, 43(W1):W65–W71, 2015.

8. J. Y. Ryu, H. U. Kim, and S. Y. Lee. Deep learning enables high-quality and high-throughput prediction of enzyme commission numbers. *Proceedings of the National Academy of Sciences*, 116(28):13996–14001, 2019.

9. H.-B. Shen and K.-C. Chou. Ezypred: a top–down approach for predicting enzyme functional classes and subclasses. *Biochemical and biophysical research communications*, 364(1):53–59, 2007.

10. C. Yu, N. Zavaljevski, V. Desai, and J. Reifman. Genome-wide enzyme annotation with precision control: Catalytic families (catfam) databases. *Proteins: Structure, Function, and Bioinformatics*, 74(2):449–460, 2009.
